# Supplementary material for: Alzheimer’s disease-related dysregulation of mRNA translation causes key pathological features with ageing
Source: Transl Psychiatry. 2020 Jun 16;10:192. doi: 10.1038/s41398-020-00882-7 (PMC7297996; doi:10.1038/s41398-020-00882-7)
Supplement: Supplementary file 1 — Supplementary Information [file 41398_2020_882_MOESM1_ESM.docx]

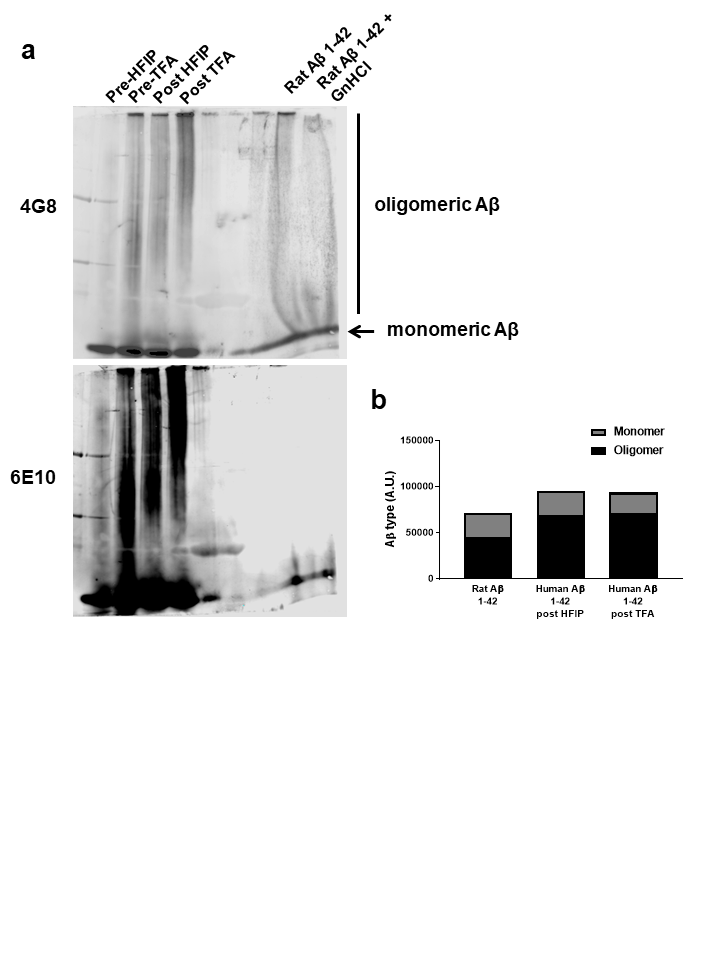


**Supplementary Figure 1.** **Composition of Aβ_1-42_ peptides used in this study, as determined by Native-PAGE.** **a** Western blot to profile Aβ_1-42_ generated. The first two lanes (“pre”) show monomeric human Aβ_1-42_ peptides solubilised in hexafluoroisopropanol (HFIP) or trifluoroacetic acid (TFA). The second two lanes (“post”) show these peptides following evaporation of solvent and oligomerisation in PBS at 37°C for 3 hours. The last two lanes show synthetic rat Aβ_1-42_ peptides prepared as detailed, before and after treatment with guanidine hydrochloride (GnHCl) which breaks down oligomers. The anti-4G8 (Aβ, aa 17-24) antibody is able to detect both the human and rat peptides, whereas the anti-6E10 (Aβ, aa 1-16) antibody can only detect the human peptide due to the sequence differences between species in this region of the peptide. **b** Quantification of the ratio of monomers to oligomers produced by the three different methods as detected by the anti-4G8 antibody, showing that the method used in this study with rat Aβ_1-42_ peptides generates fewer oligomers compared to monomers than standard methods using the human Aβ_1-42_ peptide. Only rat peptides were used in this study.


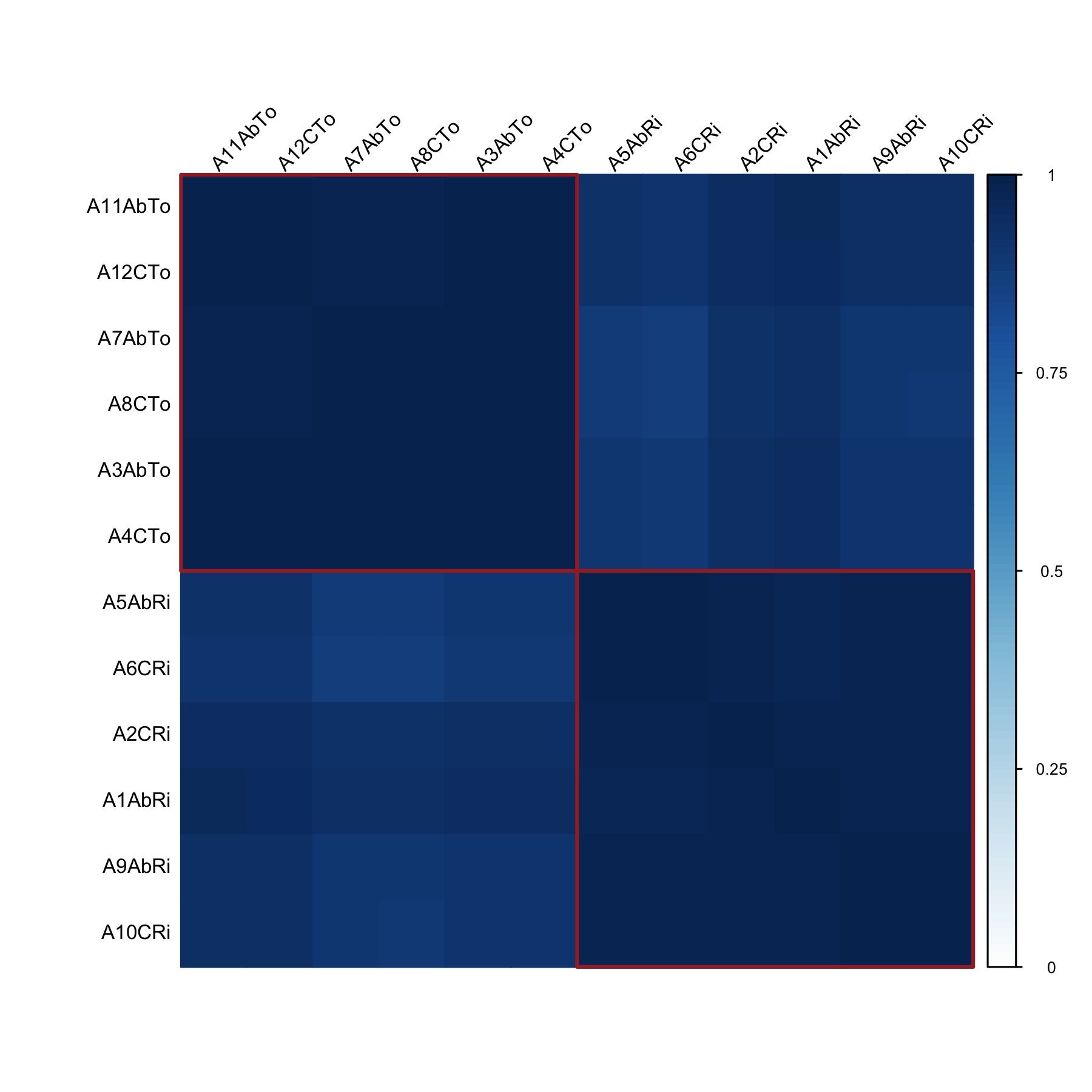


**Supplementary Figure 2.** Correlation plot showing a closer correlation within total mRNA samples and ribosome-enriched samples, in a subcellular fraction-dependent manner. This suggests that the ribosomal enrichment protocol results in distinct mRNA populations which are similar across different biological replicates. All mRNAs detected (FPKM) were employed in generating these plots.


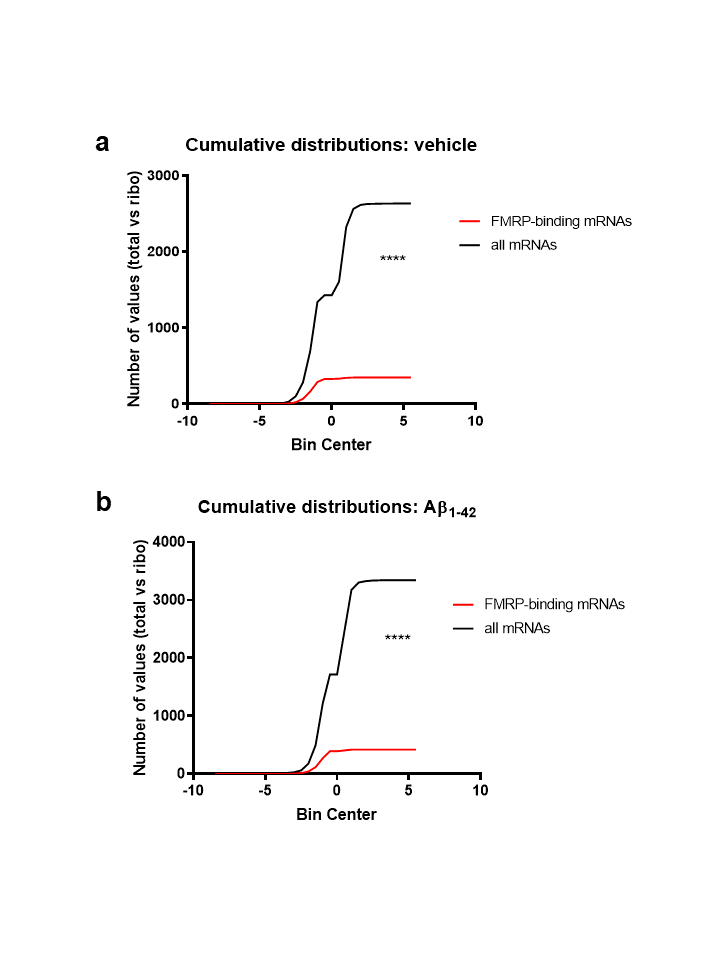


**Supplementary Figure 3.** **FMRP targets are present in both vehicle and Aβ_1-42_-treated cells. a** and **b** Cumulative distributions of mRNA changes of ‘Total’ versus ‘Ribo’ in vehicle (**a**) and Aβ_1-42_ (**b**) for FMRP versus non-FMRP targets. These graphs show that the prediction of FMRP binding causes a change in the distribution of mRNA changes between total and ribosome-enriched fractions in both vehicle- and Aβ-treated cells. **** *p* < 0.0001.


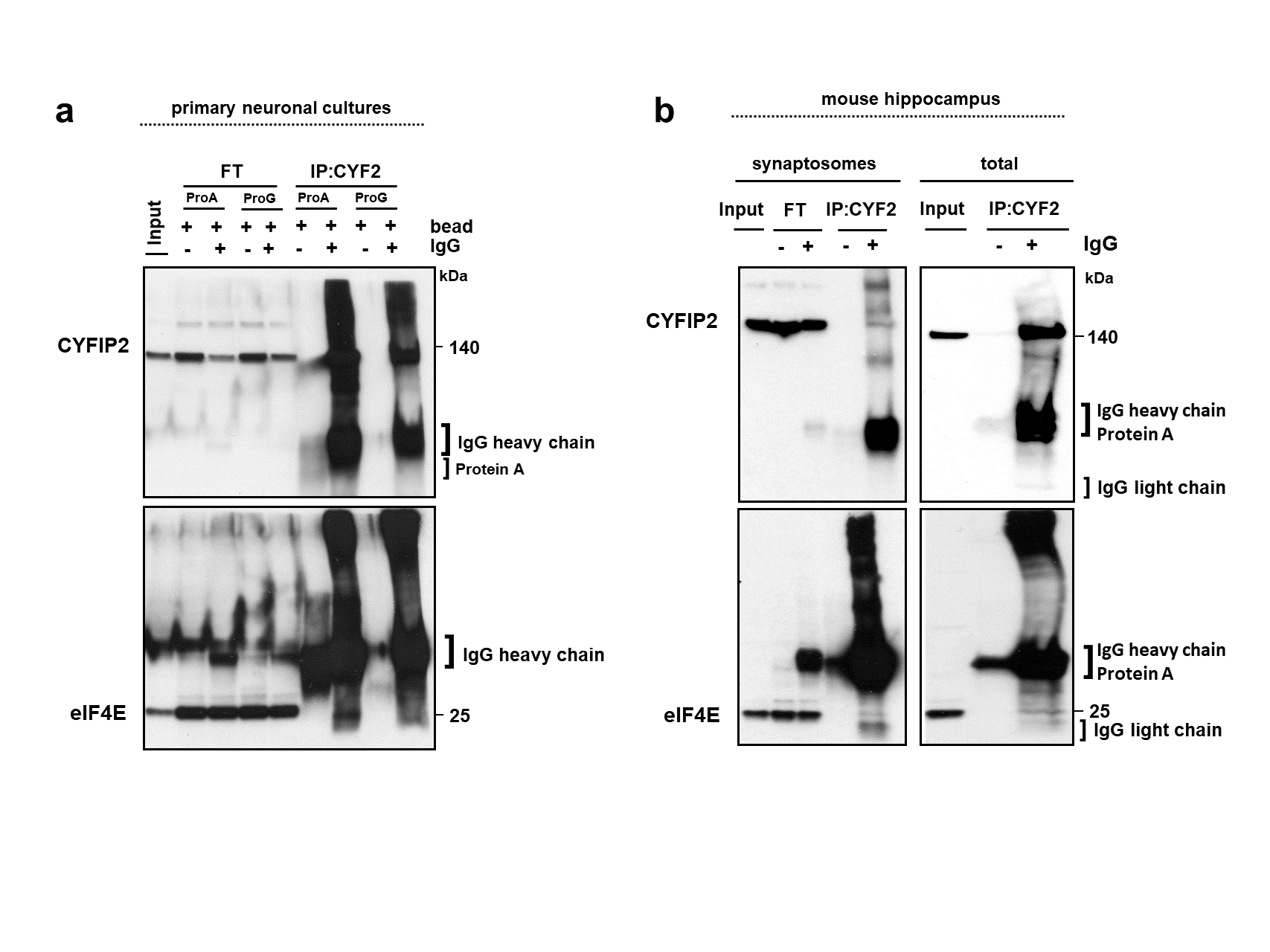


**Supplementary Figure 4.** **CYFIP2 co-immunoprecipitates with eIF4E in rat primary cortical neurons and in the mouse hippocampus.** **a** Blot showing immunoprecipitation of CYFIP2 using a primary antibody against rabbit CYFIP2 (IgG) in lysates prepared from rat primary cortical neurons at 28 DIV. Beads covalently coupled with either Protein A (**ProA**) or Protein G (**ProG**) were tested to determine efficacy. The input, FT, and IP fractions were visualised by immunoblotting for CYFIP2 and eIF4E. **b** Blots showing immunoprecipitation of CYFIP2 using the same primary antibody against rabbit CYFIP2 (IgG) in either the crude synaptosomal fraction (left) or in the total homogenate (right) prepared fresh from the mouse hippocampus. Protein A-coupled beads were used, and the input, FT, and IP were visualised by immunoblotting for CYFIP2 and eIF4E. **FT** flow-through, **IP** immunoprecipitate.


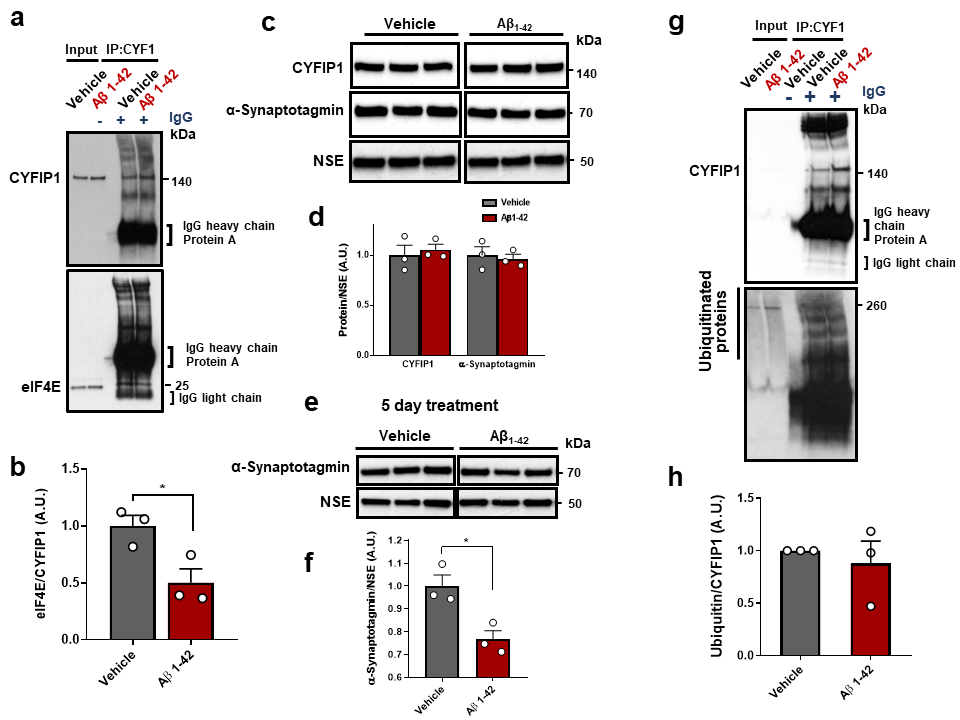


**Supplementary Figure 5.** **Aβ_1-42_ treatment results in dissociation between CYFIP1 and eIF4E but does not change CYFIP1 levels**. **a** Blots showing immunoprecipitation of CYFIP1 using a primary antibody against rabbit CYFIP1 (IgG) in lysates prepared from rat primary cortical neurons at 28 DIV, treated for 24 hours with vehicle or 100 nM Aβ1-42. The input, FT, and IP fractions were visualised by immunoblotting for CYFIP1 and eIF4E. **b** Quantification of eIF4E bound to CYFIP1 pulled down in the IP fraction in vehicle- and Aβ_1-42_-treated cells (t=3.24, p<0.05). **c** Blots showing levels of CYFIP1, α-synaptotagmin and NSE in 28 DIV primary cortical neurons after 24-hour treatment with vehicle or Aβ_1-42_ at 100 nM. **d** Quantification of CYFIP1 (t=0.46, p=0.67) and α-synaptotagmin (t=0.4, p=0.71) normalised to levels of NSE. **e** Blots showing levels of α-synaptotagmin and NSE in 28 DIV primary cortical neurons after 5-day treatment with vehicle or Aβ_1-42_ at 100 nM. **f** Quantification of α-synaptotagmin normalised to levels of NSE (t=3.8, p<0.05). **g** Blots showing immunoprecipitation of CYFIP1 in primary cortical neurons treated for 24 hours with vehicle or 100 nM Aβ_1-42_. The input, FT, and IP fractions were visualised by immunoblotting for CYFIP1 and ubiquitin. **h** Quantification of ubiquitinated proteins normalised to CYFIP1 pulled down in the IP fraction in vehicle- and Aβ_1-42_-treated cells (t=0.23, p=0.84). Data represent mean ± SEM. n=3 biological replicates (individual experiments shown as white dots). * *p* < 0.05. **FT** flow-through, **IP** immunoprecipitate.


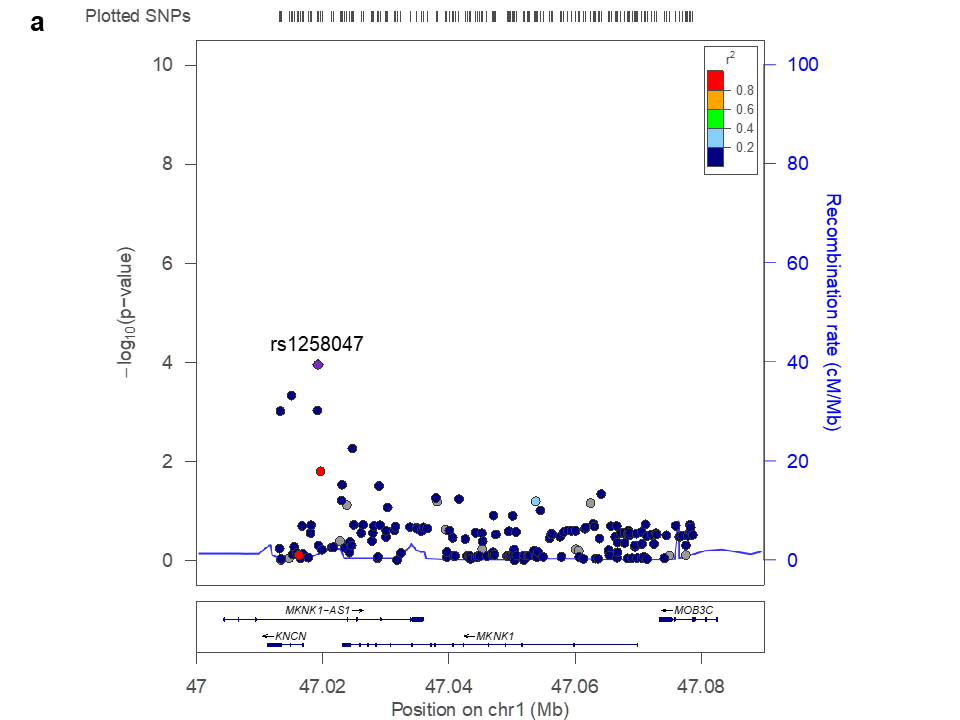

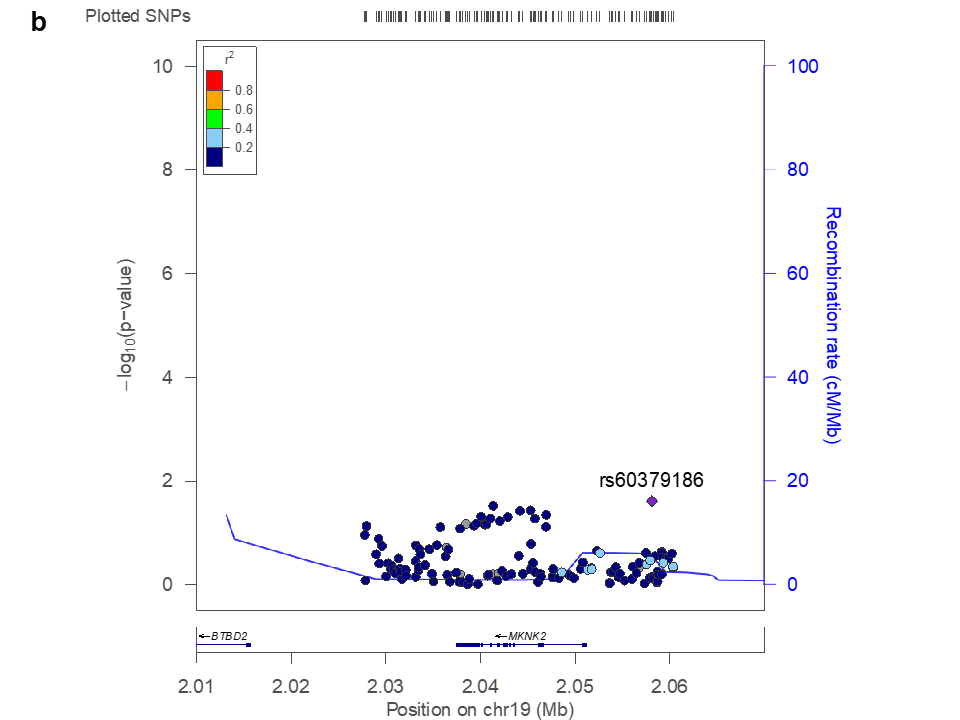

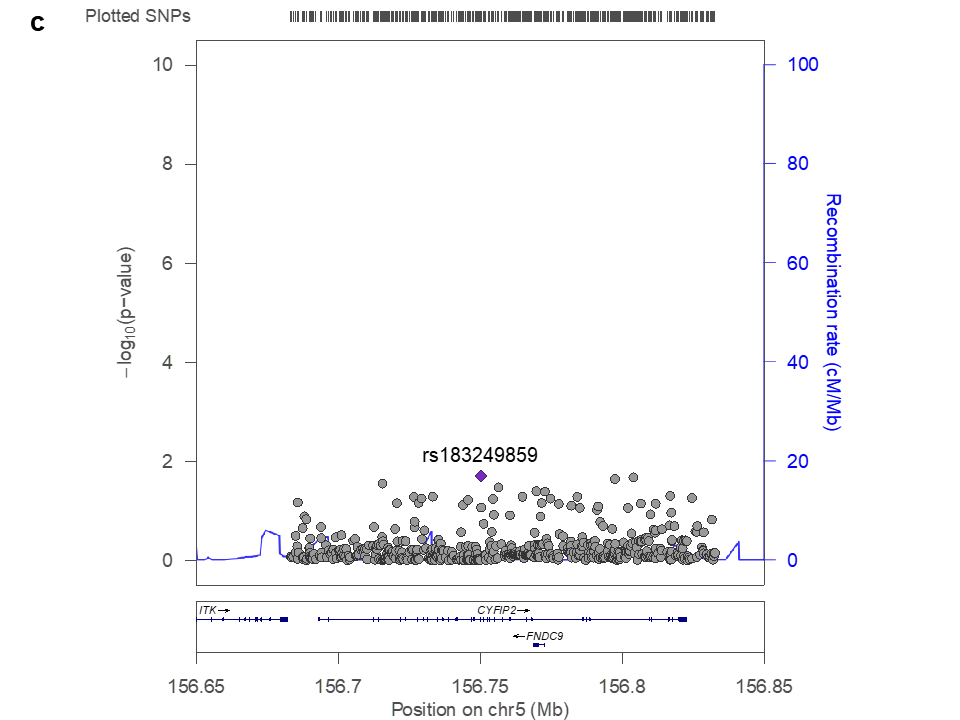

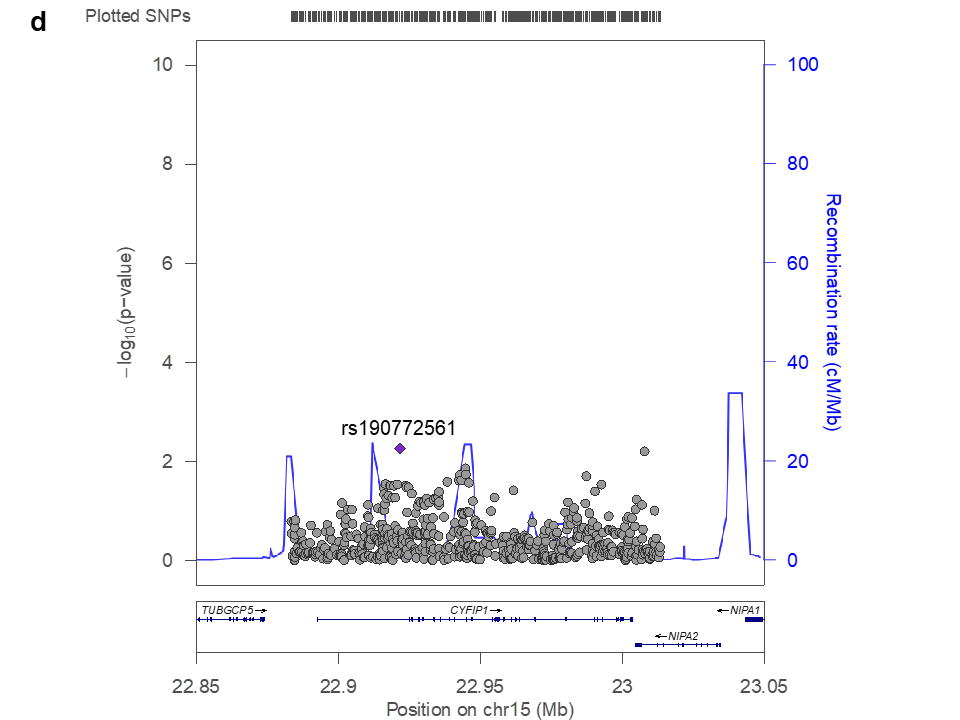

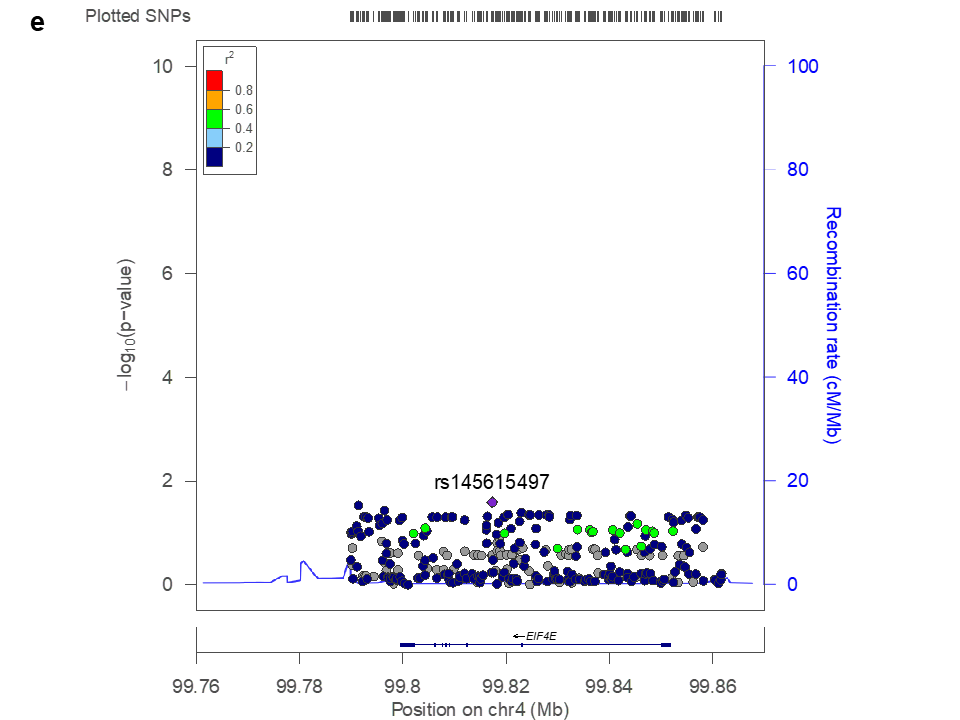


**Supplementary Figure 6. Plots showing single nucleotide polymorphisms (SNPs) within five genes, *MKNK1*, *MKNK2*, *CYFIP2*, *CYFIP1*, and *EIF4E*, tested for association with Alzheimer’s disease (AD).** No SNPs were associated with AD following correction for multiple testing. The strongest association was observed with SNP rs1258047 ~6.3 kb downstream of *MKNK1* (OR=1.474, 95% CI: 1.21 - 1.79, p=0.0001, Q=0.075). No usable LD information was available for the top SNP for *CYFIP1* and *CYFIP2* SNPs (shown as grey dots).


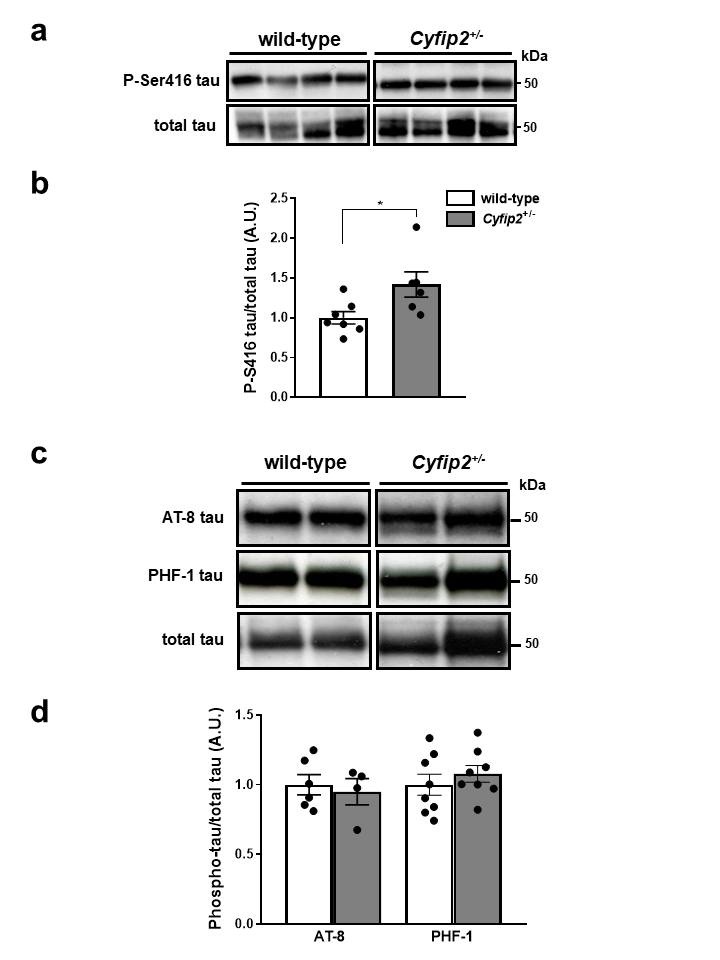


**Supplementary Figure 7. Tau phosphorylation at Ser416, but not at AT-8 or PHF-1 sites, is increased in hippocampal synaptosomes of 3-4-month-old *Cyfip2*^+/-^ mice.** **a** Representative blots of phospho-Ser416 tau and total tau in synaptosomes prepared from hippocampal tissue from 3-4-month-old *Cyfip2*^+/-^ mice and wild-type littermates. **b** Quantification of phospho-Ser416 levels normalised to total tau reveals a significant increase in young, adult *Cyfip2*^+/-^ mice (t=2.38, p<0.05). **c** Representative blots of AT-8 and PHF-1 phospho-tau and total tau in synaptosomes prepared from hippocampal tissue from *Cyfip2*^+/-^ and wild-type mice. **d** Quantification of AT-8 (t=0.42, p=0.69) and PHF-1 (t=0.8, p=0.44) levels normalised to total tau in the synaptosomal fraction does not detect a difference between genotypes. Data represent mean ± SEM. n=4-8 animals per condition (individual animals shown as black dots). * *p* < 0.05.


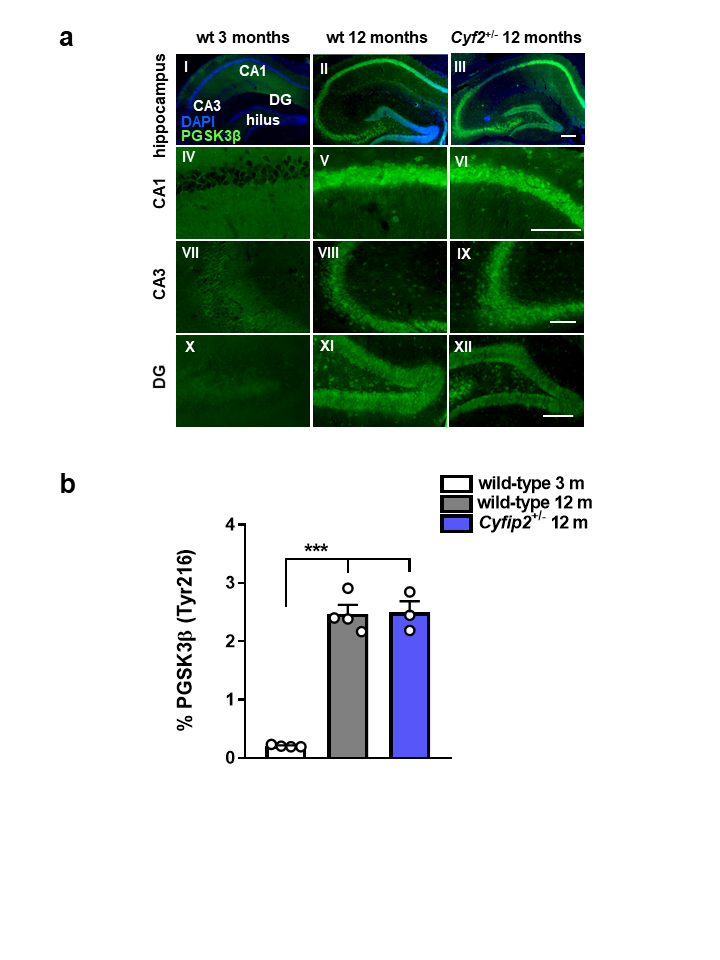


**Supplementary Figure 8. Phosphorylation of GSK3β at Tyr216 is increased in the mouse hippocampus with ageing.** **a** Representative images of dorsal hippocampal sections (**I-III**) from wild-type mice at 3 months and 12 months, and *Cyfip2*^+/-^ mice at 12 months, stained for GSK3β phosphorylated at Tyr216. Higher magnification images of CA1 (**IV-VI**), CA3 (**VII-IX**) and DG (**X-XII**). Scale bars are 200 µm. **b** Quantification of phosphoGSK3β (Tyr216) across the entire dorsal hippocampus demonstrates that this phosphorylation event is increased with ‘healthy’ ageing (q=17.71, p<0.001) but not changed further in *Cyfip*2^+/-^ mice (q=0.2040, p=0.98). Data represent mean ± SEM. n=3-4 animals per condition (individual animals shown as white dots). *** *p* < 0.001.


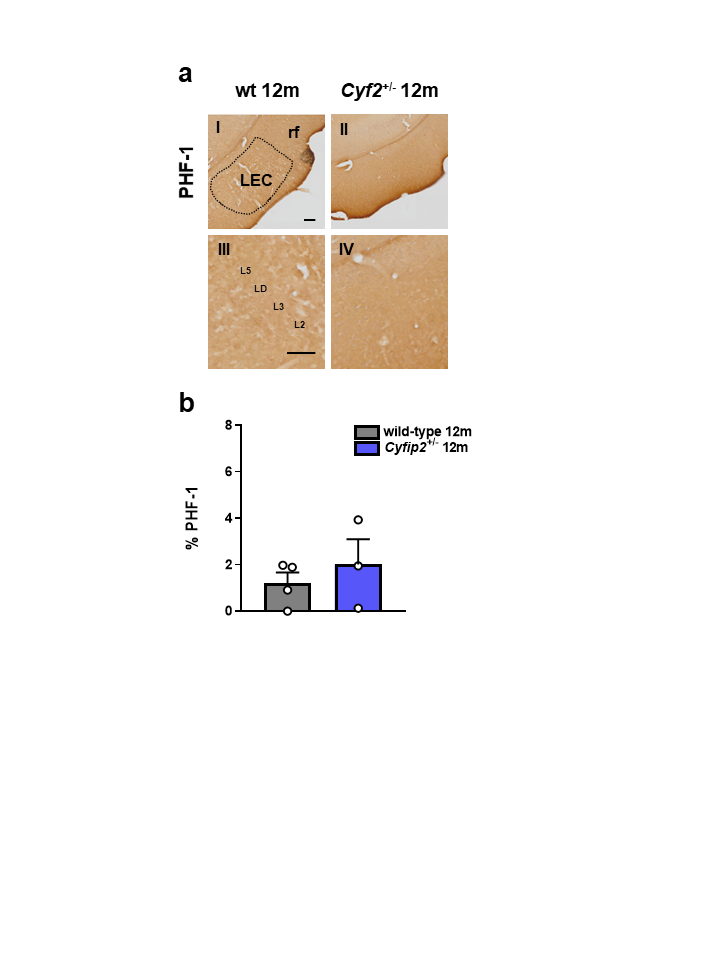


**Supplementary Figure 9. Negligible PHF-1 positive immunostaining in the entorhinal cortex of 12-month-old *Cyfip2*^+/-^ and wild-type mice.** **a** Representative images of PHF-1 staining in **LEC** of 12-month-old *Cyfip2*^+/-^ and wild-type mice (**I-II**). Higher magnification images of PHF-1 in the LEC (**III-IV**). **b** Quantification of PHF-1 positive staining in LEC does not detect a difference between genotypes (t=0.67, p=0.55). Scale bars are 100 µm. Data represent mean ± SEM. n=3-4 animals per condition (individual animals shown as white dots). **L2-5** layers 2-5 **LD** *lamina dissecans* **LEC** lateral entorhinal cortex **rf** rhinal fissure.


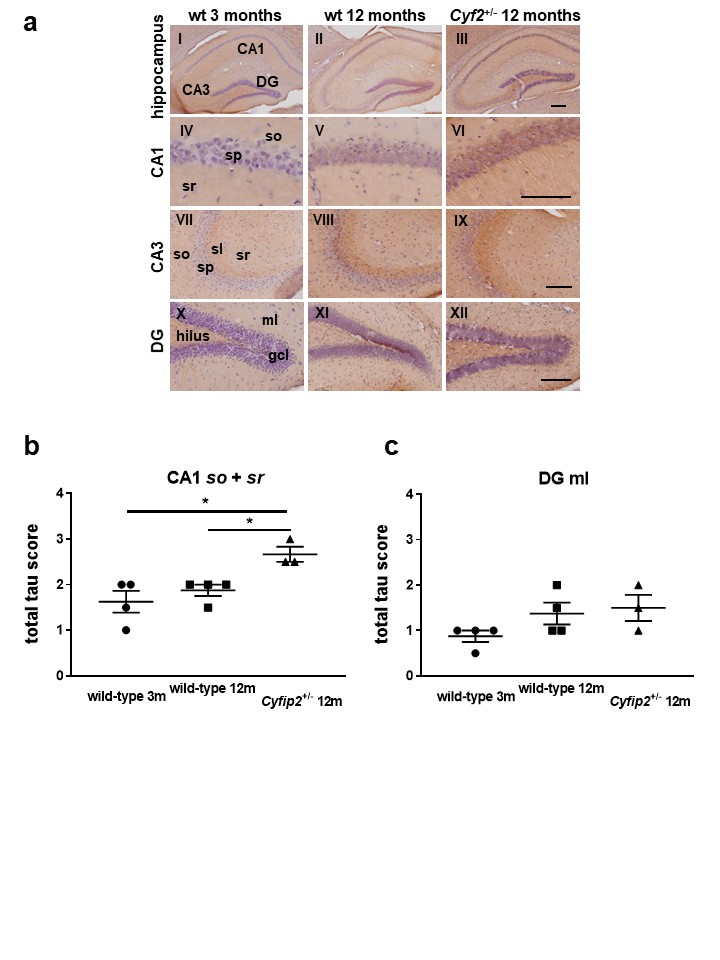


**Supplementary Figure 10. Restricted change in tau levels in 12-month-old *Cyfip2*^+/-^ mice. a** Representative images of dorsal hippocampal sections from wild-type mice at 3 months and 12 months, and *Cyfip2*^+/-^ mice at 12 months, immunohistochemically stained for total tau (**I-III**) and haemotoxylin to label nuclei. Higher magnification images of hippocampal areas CA1 (**IV-VI**), CA3 (**VII-IX**) and DG (**X-XII**). Scale bars are 200 µm. **b** Analysis of DAB staining in ***so*** and ***sr*** of area CA1 and the **ml** of the dentate gyrus indicates that tau levels are not increased in 12-month-old wild-types compared to 3-month-old wild-types (CA1 q=1.39, p=0.60; DG q=2.41, p=0.26). Tau levels are increased in 12-month old *Cyfip2*^+/-^ mice compared to age-matched wild-types in area CA1 (q=1.39, p=0.048), but not DG (q=0.56, p=0.92). Importantly, this change is much more restricted than the elevation of tau phosphorylation at AT8 and PHF-1 sites in these mutants (see, Fig. 3a, b). Data represent mean ± SEM. n=3-4 animals per condition (individual animals shown as data points). * *p* < 0.05, ** *p* < 0.01. **gcl** granular cell layer **ml** molecular layer **sl** *stratum lucidum* **so** *stratum oriens* **sp** *stratum pyramidale* **sr** *stratum radiatum*.


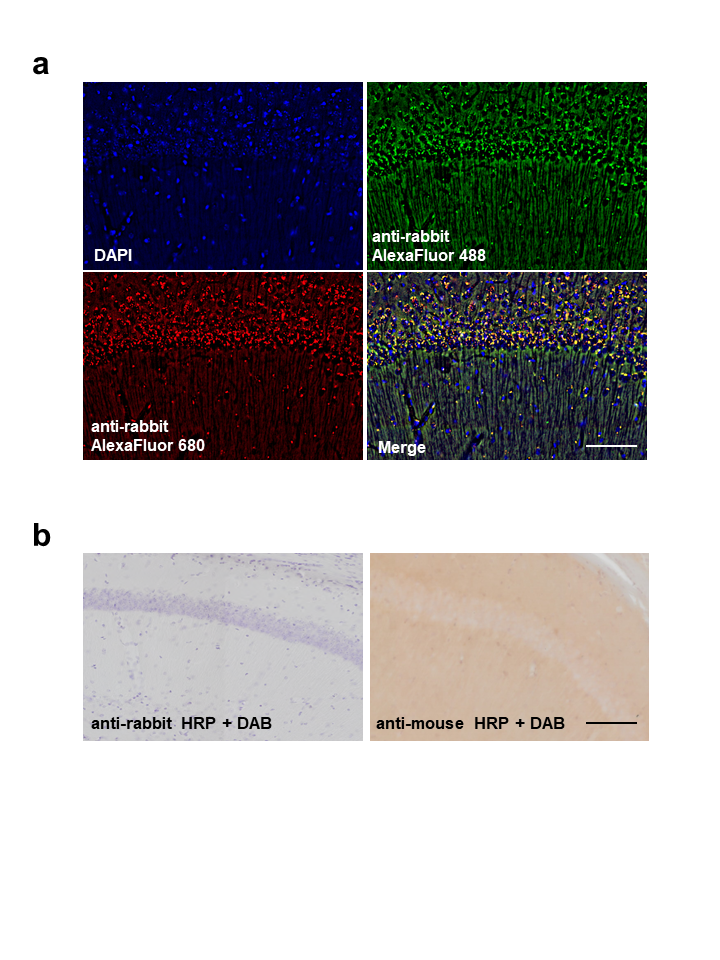


**Supplementary Figure 11.** **Staining detected in aged tissues is specific and not due to background or age-related protein build-up. a** Images of area CA1 of dorsal hippocampus from 12-month-old wild-type mice treated only with relevant fluorescent secondary antibodies and DAPI, and no primary antibodies. **b** Images of area CA1 of dorsal hippocampus from 12-month-old wild-type mice treated only with relevant HRP-tagged secondary antibodies and DAB, and counter-stained with haematoxylin, and no primary antibodies. Scale bars are 100 µm.


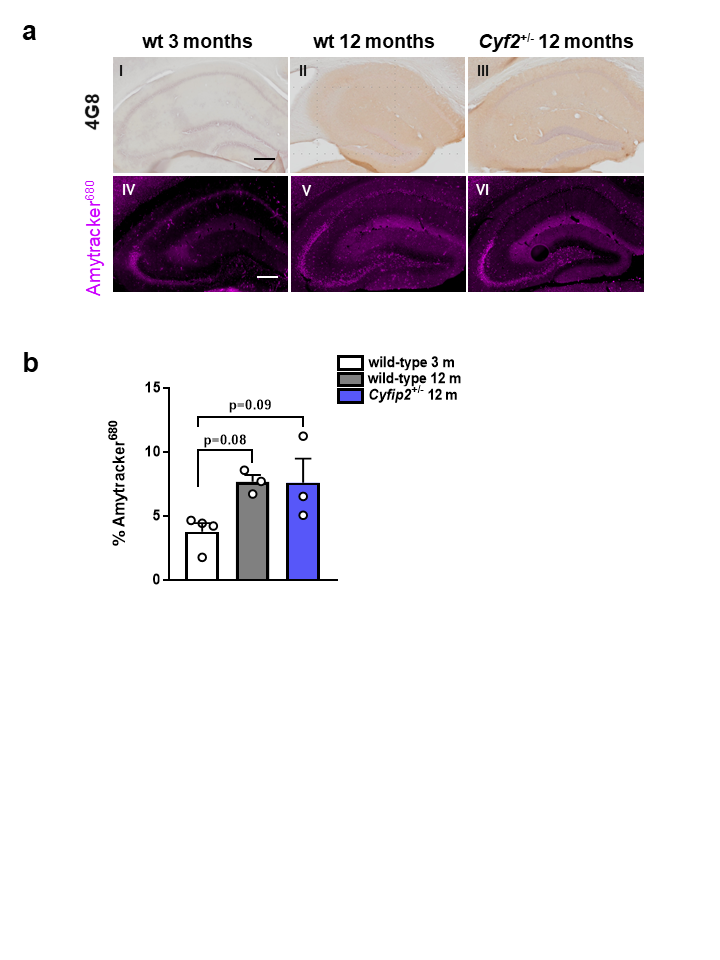


**Supplementary Figure 12. No changes in 4G8 or Amytracker^680^ staining in the hippocampus of 12-month-old wild-type and *Cyfip2*^+/-^ mice.** **a** Representative images of dorsal hippocampal sections from wild-type mice at 3 months and 12 months, and *Cyfip2*^+/-^ mice at 12 months, immunohistochemically stained for anti-4G8 antibody to detect Aβ (**I-III**) and Amytracker^680^ (**IV-VI**). Nuclei were labelled with haemotoxylin or DAPI, respectively. Scale bars are 200 µm. **b** Quantification of Amytracker^680^ staining, expressed as % of area sampled shows a trend for an increase in healthy ageing (q=3.6, p=0.08) but does not reveal a difference between aged genotypes (q=0.05, p=0.99). No 4G8-positive staining was detected across groups in the hippocampus, although aged tissues have higher background staining. Data represent mean ± SEM. n=3-4 animals per condition (individual animals shown as white dots).


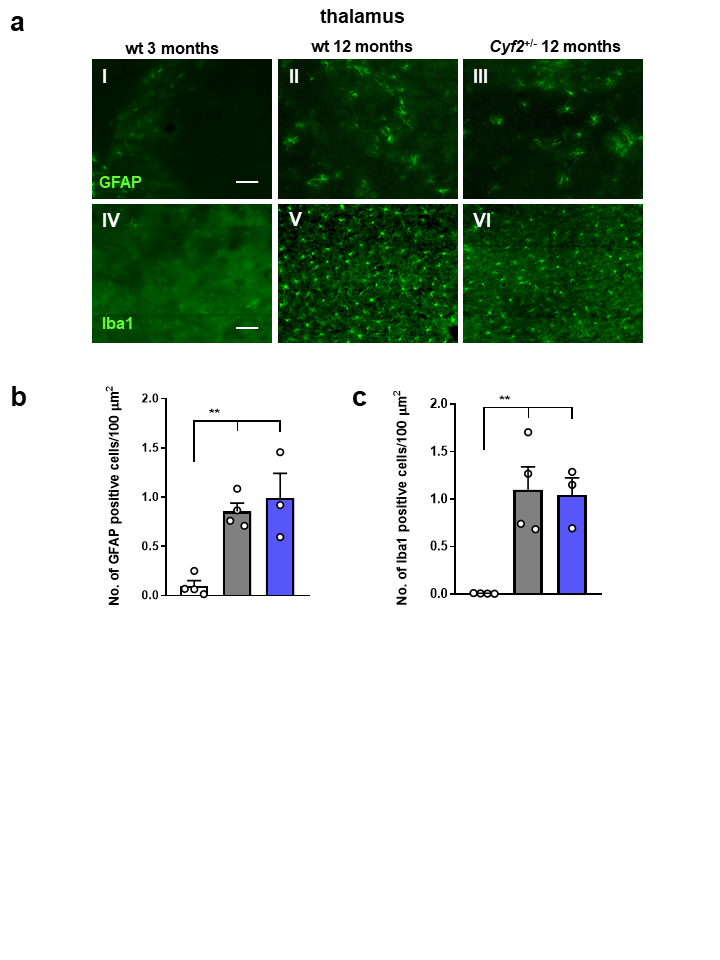


**Supplementary Figure 13. No evidence for thalamic gliosis in *Cyfip2*^+/-^ mice.** **a** Representative images of thalamic regions from wild-type mice at 3 months and 12 months, and *Cyfip2*^+/-^ mice at 12 months, immunohistochemically stained for GFAP (**I-III**) or Iba1 (**IV-VI**). Scale bars are 100 µm. **b** and **c** Quantification of number of GFAP-positive cells (**b**) and number of Iba1-positive microglia (**c**) in the mouse thalamus shows increased numbers of GFAP-positive astrocytes (q=6.07, p<0.01) and Iba1-positive microglia (q=6.54, p<0.01) in 12-month-old wild-type mice compared to 3-month-old wild-type mice, but no further changes in astrocyte and microglial numbers (GFAP q=1.01, p=0.76; Iba1 q=0.31, p=0.97) in the thalamus of 12-month-old *Cyfip2*^+/-^ mice. Data represent mean ± SEM. n=3-4 animals per condition (individual animals shown as white dots). * *p* < 0.05, ** *p* < 0.01, *** *p* < 0.001.

**Supplementary Tables 1-3 provided as Excel files.**

**Supplementary Table 1.** RNA sequencing information of samples used for analysis of total and ribosomal RNAs following Aβ_1-42_ treatment.

**Supplementary Table 2.** Log2 fold changes and corresponding p-values for transcripts showing statistically significant changes in expression between total and ribosomal fractions, under either control conditions or following Aβ_1-42_ exposure. The first two tabs display non-FMRP-binding targets, and the second two tabs display FMRP-binding targets.

**Supplementary Table 3.** Pathway analysis of genes differentially regulated in ribosomes under control conditions and following Aβ_1-42_ exposure.

**Supplementary Table 4.** Table of SNPs in five genes of interest following exclusion of SNPs in high linkage disequilibrium (LD).

| **Gene** | **Chr** | **Start*** | **End*** | **N SNPs before pruning** | **N SNPs after pruning** | **Best SNP** | **Best SNP position** | **Best SNP type** | **Best SNP**  **P-Value** | **Best SNP**  **Q-Value**** |
| --- | --- | --- | --- | --- | --- | --- | --- | --- | --- | --- |
| *MKNK1* | 1 | *4023090* | *47082515* | 205 | 85 | rs1258047 | 47019254T>C | MKNK1-AS1: Intronic  KNCN:2KB Upstream Variant | 0.0001 | 0.075 |
| *MKNK2* | 19 | *2037470* | *2051243* | 144 | 89 | rs60379186 | 2058122A>G | Upstream MNK2 | 0.024 | 0.705 |
| *CYFIP1* | 15 | 22892667 | 23003603 | 675 | 308 | rs190772561 | 22921657C>T | Intronic | 0.006 | 0.705 |
| *CYFIP2* | 5 | *156693091* | *156822606* | 593 | 201 | rs183249859 | 156750164G>C | Intronic | 0.020 | 0.704 |
| *EIF4E* | 4 | *99799607* | *99851786* | 253 | 80 | rs145615497 | 99817301C>G | Intronic | 0.025 | 0.707 |

*Gene coding region (GRCh37/hg19).

**Q-value was obtained using the FDR tools package in R.
